# Unraveling Immunotherapy Resistance in Solid Tumors: Decoding Mechanisms and Charting Future Therapeutic Landscapes

**DOI:** 10.32604/or.2025.067592

**Published:** 2025-11-27

**Authors:** Huan Wang, Jindong Xie, Na Li, Qianwen Liu, Wenqi Song, Wenkuan Chen, Cheng Peng, Hailin Tang

**Affiliations:** 1 State Key Laboratory of Oncology in South China, Guangdong Provincial Clinical Research Center for Cancer, Sun Yat-sen University Cancer Center, Guangzhou, 510060, China; 2 State Key Laboratory of Southwestern Chinese Medicine Resources, Chengdu University of Traditional Chinese Medicine, Chengdu, 611137, China

**Keywords:** Solid tumor, immune checkpoint inhibitor, ICI, drug resistance, tumor microenvironment, TME

## Abstract

Solid tumors comprise the majority of the global cancer burden, with their incidence and associated mortality posing considerable challenges to public health systems. With population growth and aging, the burden of these tumors is anticipated to increase further in the coming decades. The progression of solid tumors depends on dynamic interactions between malignantly transformed cells and the tumor microenvironment (TME). Immune checkpoint inhibitor therapy improves T cell-mediated antitumor activity by suppressing regulatory pathways, such as programmed cell death protein 1/programmed death-ligand 1. Nonetheless, its widespread application is constrained by drug resistance. In this comprehensive review, we elucidate the latest advances in understanding the mechanisms underlying drug resistance, explore pioneering approaches, such as combination therapeutic regimens and nanoscale drug delivery platforms, and propose future avenues for research. These include investigating the intricacies of drug resistance pathways, refining combination therapy strategies, and modulating the TME, along with other key areas.

## 1 Introduction

Solid tumors represent a large component of the global cancer burden [1,2]. For example, liver cancer accounted for approximately 905,700 new cases and 830,200 deaths worldwide in 2020, ranking among the leading causes of cancer-related mortality [3]. Similarly, in 2018, esophageal cancer accounted for over 572,000 new cases globally, with esophageal adenocarcinoma and esophageal squamous cell carcinoma being the predominant subtypes [4]. Furthermore, colorectal cancer ranks as the third most common malignancy globally, with a marked increase in incidence reported among the working-age population in 2021 [5]. The age-standardized incidence rate (ASIR) and age-standardized mortality rate (ASMR) for certain solid tumors have declined; however, the absolute number of cases continues to rise. For instance, by 2040, the number of liver cancer cases is projected to increase by 55% [3].

Solid tumors, defined as tissue masses originating from abnormal cellular proliferation, are frequently detected in organs, such as the lungs, breasts, and prostate glands [1]. These tumors demonstrate substantial clinical heterogeneity, and their pathogenesis involves complex mechanisms, including the accumulation of genetic mutations, epigenetic dysregulation, and the dynamic modulation of tumor microenvironment (TME). Surgical excision, radiotherapy, and chemotherapy form the foundation of existing therapeutic strategies. Nonetheless, challenges, such as drug resistance and postoperative recurrence, persist, with only marginal improvements observed in 5-year survival rates for patients with advanced-stage disease [6]. Despite the partial success of conventional therapies, including surgery, radiotherapy, and chemotherapy, drug resistance and tumor relapse continue to pose major challenges.

Immune checkpoint inhibitors (ICIs) have revolutionized cancer therapy, markedly enhancing treatment outcomes across several malignancies. By inhibiting immunosuppressive signaling pathways, ICIs restore T cell-mediated cytotoxicity against tumors, offering an innovative therapeutic option for solid tumors [7]. Notably, where conventional chemotherapy and targeted therapies fail, ICIs have exhibited exceptional clinical efficacy [8]. Nonetheless, the intricate immunosuppressive network within the TME contributes to overall response rates of less than 30%. Both primary resistance (absence of an initial therapeutic response) and acquired resistance (loss of efficacy after initial success) are intricately associated with tumor heterogeneity, antigen loss, and myeloid cell-mediated immunosuppression [9]. To this end, ongoing investigations focus on innovative combination strategies, such as combining ICIs with anti-angiogenic agents or enhancing tumor-specific recognition through engineered T cell receptors [10]. These cutting-edge approaches are undergoing rigorous clinical evaluation and show promise for overcoming existing therapeutic limitations. This article synthesizes current breakthroughs in elucidating drug resistance mechanisms and highlights promising avenues for future investigation.

## 2 Mechanisms of Immune Checkpoint Resistance

As a landmark advancement in cancer therapeutics, ICIs restore T-cell antitumor activity by obstructing signaling pathways, notably programmed cell death protein 1/programmed death-ligand 1 (PD-1/PD-L1) and cytotoxic T-lymphocyte-associated protein 4 (CTLA-4) [7]. Two underlying mechanisms are involved: (i) binding of PD-1 receptors to PD-L1 ligands on T cells, which are overexpressed on tumor cells, leading to T-cell exhaustion; (ii) CTLA-4 competitively binds to cluster of differentiation (CD)80/CD86 co-stimulatory molecules, thereby inhibiting early-stage T-cell activation [11]. Despite clinical improvements in some patients receiving ICIs, most of them demonstrate limited therapeutic efficacy [12]. Notably, objective response rates (ORRs) range between 40% and 70% for malignancies, including microsatellite instability-high tumors, Hodgkin lymphoma, and melanoma, whereas most other cancer types demonstrate ORRs as low as 10% to 25% [13]. Importantly, even patients who initially respond to treatment may develop acquired secondary resistance, leading to disease progression [14].

### 2.1 Tumor-Intrinsic Mechanisms

**Figure 1:**
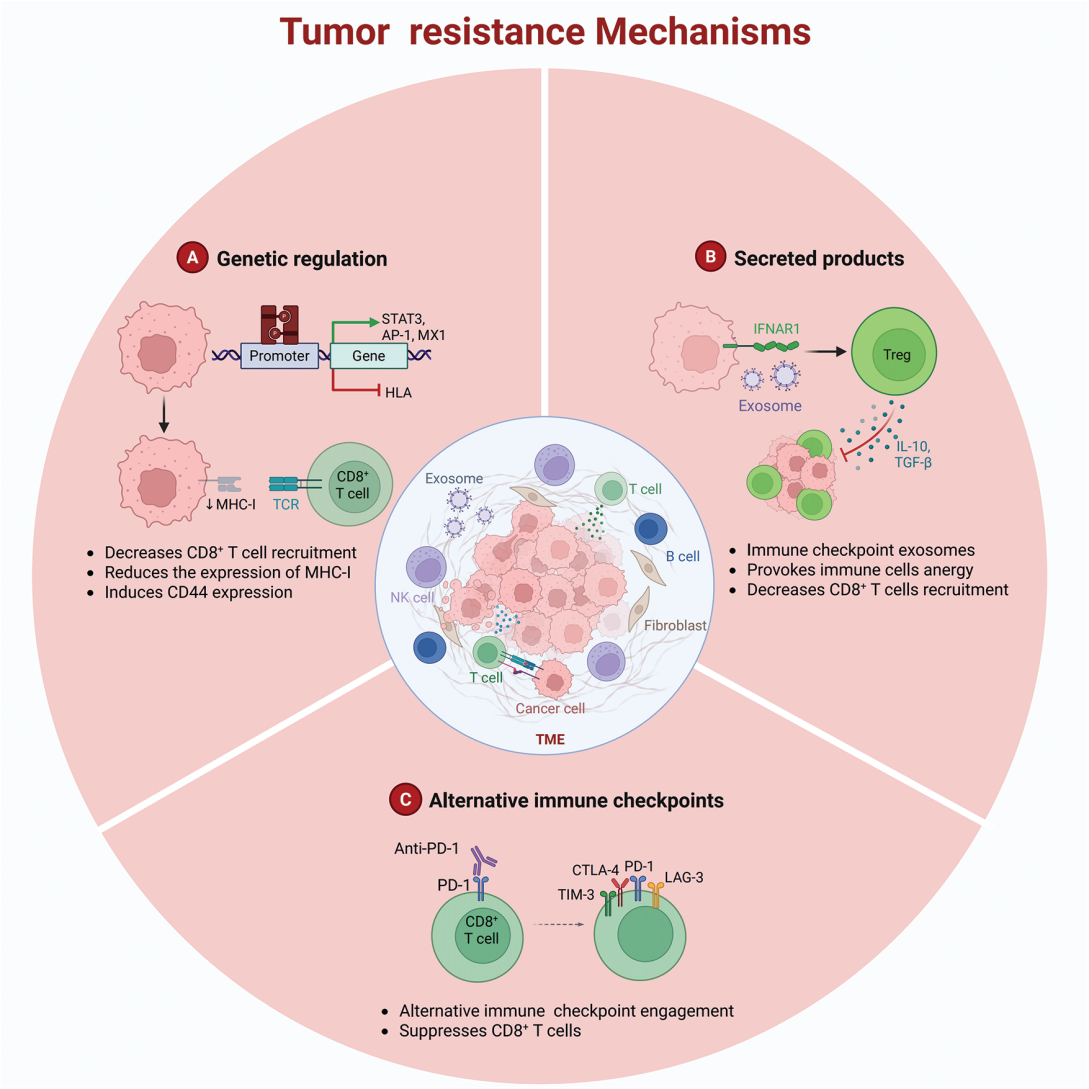
Tumor resistance mechanisms (Created using Biorender)

A fundamental mechanism underlying drug resistance is the capacity of tumor cells to facilitate immune evasion through dynamic genetic alterations [15]. This strategy is characterized by two key processes: (i) downregulation of antigen presentation-related gene expression via mutations or epigenetic modifications (Fig. 1A) and (ii) upregulation of immunosuppressive molecule expression. For example, in ovarian cancer models, epigenetic silencing controlled by enhancer of zeste homolog 2-induced histone H3 lysine 27 trimethylation and DNA methyltransferase 1-mediated DNA methylation suppresses the expression of T helper 1-type chemokines C-X-C motif chemokine ligand 10 and C-X-C motif chemokine ligand 9. This suppression decreases tumor-infiltrating T cell density, thereby reducing the effectiveness of PD-L1 inhibitors.

Moreover, primary resistance can originate from inadequate tumor antigenicity or defective antigen presentation. For example, the downregulation of major histocompatibility complex class I molecules is caused by mutations in phosphatidylinositol 3-kinase/protein kinase B and mitogen-activated protein kinase pathways [16]. Through epigenetic silencing (e.g., promoter methylation) or genetic alterations (e.g., β2-microglobulin mutations), tumor cells substantially reduce their antigen-presenting capacity, preventing recognition of tumor-associated antigens by T cells [17].

### 2.2 Immunosuppressive Remodeling of the TME

Immune cells residing in the TME demonstrate considerable heterogeneity [18]. Immunosuppressive cell populations, including myeloid-derived suppressor cells (MDSCs), regulatory T cells (Tregs), and M2-type tumor-associated macrophages, release inhibitory cytokines, such as interleukin-10 and transforming growth factor-beta, which impair effector T-cell activity and contribute to drug resistance (Fig. 1B) [19]. MDSCs further exacerbate immunosuppression by suppressing the activation and expansion of natural killer cells and effector T cells, inducing Treg differentiation, and impairing the antigen-presenting capacity of antigen-presenting cells (APCs) [20]. Subsequently, they facilitate immune evasion [21].

Another substantial resistance mechanism is the compensatory activation of immune checkpoints. Beyond the PD-1/PD-L1 pathway, co-inhibitory molecules, such as CTLA-4, lymphocyte activation gene 3 (LAG-3), and T cell immunoglobulin and mucin-domain containing-3 (TIM-3), attenuate T-cell function, thereby limiting the effectiveness of immune checkpoint therapies (Fig. 1C) [22]. Both TIM-3 and PD-1 modulate immune responses by inhibiting T cell functionality. TIM-3 is an activation-induced inhibitory receptor that promotes T cell exhaustion and immune tolerance by interacting with ligands, such as galectin-9 and carcinoembryonic antigen-related cell adhesion molecule 1 [23]. Conversely, PD-1 attenuates T cell activation and proliferation by binding to PD-L1 or programmed death-ligand 2, thereby preserving immune homeostasis [24]. Importantly, TIM-3 and PD-1 can be co-expressed on exhausted T cells under specific conditions. This co-expression amplifies inhibitory signaling and synergistically suppresses T cell activity via distinct molecular mechanisms [25]. Within the TME, this co-expression facilitates tumor immune evasion. For example, in hepatocellular carcinoma and acute myeloid leukemia, the co-expression of TIM-3 and PD-1 is strongly related to T cell exhaustion and tumor progression [26].

Compensatory upregulation of TIM-3, LAG-3, or T cell immunoreceptor with Ig and ITIM domains occurs after PD-1 blockade. For example, in murine lung adenocarcinoma models, acquired resistance and tumor progression after an initial anti-PD-1 response were strongly related to increased TIM-3 expression. Sequential targeting of TIM-3 with blocking antibodies significantly enhanced survival rates. Moreover, the overexpression of CTLA-4 and LAG-3 on T cells has been observed in tumors showing resistance [27].

The TME encompasses the extracellular matrix (ECM), a noncellular component that provides essential biochemical signals and structural support to tumor cells [28]. Beyond functioning as a structural scaffold, the ECM participates in immune modulation and drug resistance. Its biomechanical properties and biochemical signaling can influence the immune microenvironment. For example, decellularized ECM can induce M2 macrophage polarization by optimizing its micromechanical properties, promoting anti-inflammatory responses and tissue rapid [29]. Additionally, the ECM can hinder drug diffusion and reduce treatment efficacy by increasing mechanical stiffness and altering its composition. Structural abnormalities in tumor ECM create physical barriers to drug delivery, fostering drug resistance [30]. Cancer-associated fibroblasts (CAFs) promote therapeutic resistance by remodeling the ECM to support tumor cell proliferation and survival while restricting immune cell infiltration and drug delivery. In pancreatic cancer, CAF-mediated excessive ECM deposition creates a dense stromal barrier that limits drug accessibility and induces chemoresistance [31].

The mechanisms by which tumors develop drug resistance may involve the following three aspects: A. Downregulation of antigen presentation-related gene expression via mutations or epigenetic modifications; B. Immunosuppressive cell populations releasing interleukin-10 and transforming growth factor-β, among others, leading to immune cell anergy; C. Compensatory activation of immune checkpoints resulting in reduced efficacy of immune checkpoint therapies.

## 3 Breakthrough Developments

### 3.1 Strategies of Combination Therapy

ICIs have achieved notable success in the treatment of solid tumors; nevertheless, the emergence of drug resistance remains a considerable challenge in clinical practice. To this end, combination therapies have emerged as a crucial approach, integrating diverse treatment modalities to synergistically enhance antitumor immune responses and mitigate drug resistance. The integration of chemotherapy with ICIs offers distinct advantages: chemotherapeutic agents directly destroy tumor cells while improving immune response by releasing tumor antigens and increasing tumor immunogenicity. This dual effect addresses the limitations of monotherapy by augmenting antigen presentation and enhancing tumor immunogenicity [32].

Likewise, the integration of targeted therapy with ICIs emphasizes precision-based therapeutic interventions. Targeted therapies inhibit tumor proliferation by blocking specific signaling pathways, whereas ICIs enhance systemic immune responses. Novel antibody-drug conjugates (ADCs), exemplifying a class of targeted agents, have demonstrated considerable potential in preclinical and clinical trials when combined with ICIs. They leverage antibody-mediated precision delivery mechanisms [33]. Radiotherapy, a localized treatment approach, works synergistically with ICIs by inducing radiation-induced immunogenic cell death (ICD). This mechanism not only controls local tumor lesions but also initiates systemic immune responses. In solid tumors, such as melanoma, combination therapy has enhanced immune cell infiltration and antigen release [34].

Immunomodulators and dual immune checkpoint blockade strategies broaden the scope of combination therapy. Tumor vaccines combined with ICIs specifically improve T-cell activation. The simultaneous inhibition of PD-1 and CTLA-4 has shown superior performance in tumors with high immunogenicity [35]. Interventions targeting the TME are equally vital. Anti-angiogenic agents ameliorate abnormal tumor vasculature and inhibit immunosuppressive cells, creating a favorable milieu for immunotherapy. Epigenetic modulators, such as histone deacetylase inhibitors, augment tumor immunogenicity by altering gene expression profiles, and their combination with ICIs has demonstrated synergistic effects in treatment-resistant tumors [32].

Recent advances in combination therapies have introduced innovative therapeutic strategies. A novel electroimmunotherapy combined with chimeric antigen receptor T cell (CAR-T) therapy has shown efficacy in solid tumors by inducing ICD and the remodeling of the TME. This, in turn, enhances CAR-T cell antitumor activity. Furthermore, substantial progress has been made in integrating ICIs with oncolytic viruses (OVs). The synergistic application of OVs and ICIs reshapes the immunosuppressive TME and enhances systemic immune responses [36]. Notably, preclinical studies have suggested that OVs engineered to express interleukin-12 and granulocyte-macrophage colony-stimulating factor, when combined with PD-1 blockade, exhibit robust antitumor activity even in poorly immunogenic solid tumors [37].

The combination of ICIs with other therapeutic modalities has markedly improved therapeutic outcomes. Nonetheless, it has increased the complexity and severity of adverse effects. Combination immunotherapy can precipitate a broad range of immune-related adverse events (irAEs) affecting multiple organ systems. In the gastrointestinal tract, irAEs may induce diarrhea and colitis [38]. Dermatologic manifestations include skin rashes and pruritus [39], with severe cases potentially escalating to Stevens-Johnson syndrome. Hematologic alterations, such as leukopenia and anemia, are frequently observed [40]. The incidence of all-grade adverse events escalates substantially upon combining chemotherapy with ICIs. In contrast, dual immunotherapy regimens (e.g., CTLA-4 + PD-1 inhibitors) are associated with an even higher risk of grade 3 or higher toxicities. To mitigate these adverse effects, a graded management approach, early intervention with corticosteroids, and personalized adjustments to treatment regimens, such as optimizing chemotherapy dosing, are warranted.

To summarize, combined therapeutic strategies enhance overall outcomes in solid tumors through multidimensional mechanisms. With ongoing clinical research and the advent of innovative therapies, this integrated strategy holds promise for overcoming drug resistance and improving long-term survival.

### 3.2 Potential of Nanomedicine Delivery Systems

Nanomedicine delivery systems, as emerging technological platforms, offer innovative strategies to address drug resistance. They substantially enhance the pharmacokinetic profiles of therapeutic agents via nanoparticle carriers, extending the half-life of drug circulation, improving bioavailability, and precisely modulating drug distribution patterns *in vivo*. Consequently, they enable the precise targeting of therapeutic sites. This capability allows immunotherapeutic drugs to accumulate in tumor tissues or specific immune cell regions, thereby enhancing antitumor efficacy while minimizing systemic toxicity [41].

To address resistance mechanisms in immune checkpoint therapy, nanocarrier systems offer distinct advantages through multidimensional strategies. They operate through three primary mechanisms:

1. Targeted ligand modifications facilitate drug-specific accumulation within the TME or immune cells, thereby minimizing nonspecific drug diffusion and enhancing drug concentration at lesion sites [42].
2. The sustained and controlled release capabilities of nanocarriers maintain effective drug concentrations in therapeutic areas, reducing the likelihood of resistance [43].
3. Combination strategies that co-deliver chemotherapeutic agents and immunomodulators address the limitations of monotherapy, generating synergistic antitumor immune responses [44].

Importantly, these systems extend beyond conventional drug delivery by exerting multifaceted immunomodulatory effects, such as inducing ICD to facilitate antigen release [45], enhancing the efficiency of APCs, and remodeling the TME through immunoregulatory molecule delivery.

Nanocarriers encompass a diverse array of types, each possessing unique physicochemical characteristics (Table 1). These include niosome and lipid-based formulations, polymeric, inorganic, hybrid, protein-based, and carbon-based nanoparticles. Lipid-based nanoparticles, such as liposomes, solid lipid nanoparticles, and nanostructured lipid carriers, are extensively used because of their inherent biocompatibility and biodegradability [46]. These properties improve drug solubility and bioavailability, reduce drug toxicity and adverse effects, and facilitate sustained and targeted drug delivery [47]. Nonetheless, limitations, such as stability issues during preparation and potential immune recognition and clearance *in vivo* may limit efficacy [47].

**Table 1:** Types of nanocarriers and the advantages and disadvantages of each type

Types of nanocarriers	Liposomes and niosomes	Polymeric	Inorganic	Hybrid	Protein-based	Carbon-based
Advantages	-High biocompatibility -High biodegradability -Low toxicity -Low immunogenicity -Encapsulation of hydrophilic and lipophilic drugs -Scalable manufacturing process [47]	-Easy loading of hydrophobic drugs -Low production costs compared with other nanosystems -Scalable manufacturing process [47]	-Versatility of surface functionalization -Stimuli responsive behavior -Uniformity in particle size [50]	-High stability -Controllable drug release -Low toxicity -High biocompatibility [52]	-High biocompatibility -High biodegradability -Non-immunogenicity -Non-toxic -High physical stability [52]	-High drug delivery efficiency -High biocompatibility controllable drug release -Environmental friendliness [54]
Disadvantages	-Short half-life -Possibility of hydrolysis-like oxidation -High production costs -Low stability [47]	-Low biocompatibility compared to others -Low stability -Complex characterization -Dependency of critical micelle concentration [47]	-Higher toxicity -Less biocompatibility -Poor data regarding long-term stability [50]	-Complex industrial manufacturing -High production costs [52]	-Expensive manufacturing -Complex industrial manufacturing -Lower loading capacity -Less blood circulation time [53]	-Complex industrial manufacturing -Potential biological toxicity [55]

Polymeric nanoparticles, including poly(lactic-co-glycolic acid) and polyethylene glycol [48], are widely used for sustained and targeted drug delivery. These carriers possess high biocompatibility and controlled drug release profiles, enabling prolonged drug release [49]. However, complex preparation processes and low drug encapsulation efficiency present potential drawbacks [47]. Inorganic nanoparticles, such as gold nanoparticles, carbon nanotubes, and hydroxyapatite nanoparticles, exhibit high stability, favorable mechanical properties, and multifunctionality, which makes them suitable for both drug delivery and imaging [50]. Nevertheless, certain inorganic carriers may pose biotoxicity risks and limited biodegradability [50]. Hybrid nanoparticles, such as lipid-polymer hybrids or inorganic-organic hybrids, facilitate multifunctionality, including concurrent targeted drug delivery and imaging, thereby enhancing therapeutic efficacy [51]. Despite these advantages, their synthesis involves intricate processes, and compatibility challenges may emerge between constituent materials [52].

Protein-based nanoparticles, including recombinant casein micelles, have garnered considerable research interest because of their natural origin and biocompatibility. These carriers exhibit remarkable biocompatibility and biodegradability, which facilitate effective drug encapsulation and targeted delivery [53]. Nonetheless, they are susceptible to enzymatic degradation *in vivo*, and their stability may be affected by environmental stressors [52]. In contrast, carbon-based nanoparticles, such as graphene oxide and fullerenes, are used in drug delivery applications because of their distinctive physicochemical properties, such as high specific surface areas and exceptional drug-loading capacities [54]. However, certain carbon-based carriers may present biotoxicity risks and environmental hazards [55].

The selection of nanodrug carriers requires a thoughtful consideration of the specific clinical application along with the properties of both the carrier and the drugs. Lipid-based and polymeric carriers are extensively utilized because of their biocompatibility and controllability. In contrast, inorganic and hybrid carriers are utilized in specialized applications because of their multifunctionality and high stability. With the ongoing progress in nanotechnology, the development and clinical application of innovative nanocarriers are expected to increase.

Despite their noteworthy clinical potential, nanocarrier systems face substantial challenges in translational medicine. A more comprehensive monitoring framework is warranted for the long-term biosafety assessment of nanomaterials. Currently, there are no unified regulatory guidelines regarding the critical quality attributes of nanodrugs, such as the particle size, surface charge, and stability [56]. Moreover, insufficient data on long-term biocompatibility and immunogenicity have resulted in the discontinuation of some clinical trials because of toxicity concerns [56]. Furthermore, nanocarrier systems encounter a low conversion rate from preclinical research to clinical application. Despite extensive preclinical studies, less than 5% of nanodrugs receive regulatory approval [56]. Nonetheless, with ongoing progress in nanotechnology and translational research, these cutting-edge delivery systems hold promise to enhance existing immunotherapies and introduce novel pathways to address therapeutic challenges in solid tumors.

## 4 Discussion

Immunotherapy targets immune checkpoints and has attained remarkable progress in the management of solid tumors; however, its clinical application continues to encounter substantial challenges, including drug resistance and interindividual variability in therapeutic efficacy. At the foundational research level, researchers should comprehensively elucidate the molecular mechanisms underlying ICIs, particularly emphasizing the complex network of downstream signaling pathways. Clarifying the regulatory principles governing these pathways will facilitate identifying the molecular basis for resistance mechanisms, thereby enabling the development of precise intervention strategies. Simultaneously, to overcome the limitations of monotherapy, researchers are actively investigating combination therapeutic approaches. These include integrating ICIs with CAR-T cell therapy or targeted agents [57] to enhance synergistic effects through optimized dosing ratios and administration schedules.

Personalized medicine provides novel opportunities to overcome immunotherapy resistance in solid tumors through precise diagnostic and therapeutic approaches. This field relies on the identification of biomarkers that can predict both treatment response and resistance. For example, TMEM92 is a pivotal biomarker of immune resistance in pancreatic cancer, and its elevated expression is associated with poor prognosis and suboptimal immunotherapy outcomes [58]. The identification of such biomarkers facilitates the selection of patients who are more likely to benefit from immunotherapy while enabling the development of alternative treatment regimens for patients exhibiting resistance.

Advanced single-cell multi-omics technologies, such as genomics, transcriptomics, and proteomics, provide insights into tumor heterogeneity and resistance mechanisms [59]. By examining cellular diversity within the TME, researchers can identify molecular pathways that drive resistance, thereby informing the development of targeted therapeutic strategies. These technologies facilitate personalized medicine to address tumor heterogeneity and treatment resistance [60]. Concurrently, multi-omics technologies can be synergistically integrated with artificial intelligence (AI), which shows considerable promise in predicting responses and mechanisms underlying resistance. AI can analyze multi-omics data, enabling the prediction of treatment responses and the optimization of personalized treatment plans, thereby improving both the precision and efficacy of therapeutic interventions [61].

The integration of emerging technologies has become crucial for facilitating scientific breakthroughs. Single-cell sequencing facilitates the dynamic analysis of tumor heterogeneity, whereas AI systems can predict individualized treatment responses [62]. The synergistic application of these technologies’ advances not only the discovery of biomarkers but also the development of personalized treatment regimens.

In the realm of technological innovation, researchers are exploring three avenues: (i) the ongoing identification of novel immune checkpoint molecules to broaden therapeutic targets [34]; (ii) the refinement of drug delivery systems through nanocarriers and ADCs; and (iii) the augmentation of anti-tumor immunity via metabolic reprogramming strategies. The advancement of these innovative methodologies necessitates a seamless integration with clinical translation. Upcoming clinical trials will focus on biomarker-guided patient stratification and utilize translational medicine platforms to expedite the application of laboratory discoveries into clinical practice.

## Data Availability

Not applicable.

## Abbreviations

TME: tumor microenvironment
ICI: immune checkpoint inhibitor
ORR: objective response rates
MDSCs: myeloid-derived suppressor cells
Tregs: regulatory T cells
APCs: antigen-presenting cells
ADCs: antibody-drug conjugates
ICD: immunogenic cell death
